# The role of interleukin-17 and interleukin-23 inhibitors in the development, progression, and recurrence of cancer: A systematic review

**DOI:** 10.1016/j.jdin.2024.06.006

**Published:** 2024-08-16

**Authors:** Marie Vangilbergen, Aline Stockman, Axelle Van De Velde, Maria Garmyn, Kevin Punie, Tom Hillary

**Affiliations:** a Department of Dermatology, University Hospitals Leuven, Leuven, Belgium; b Research Group of Dermatology, University of KULeuven, Leuven, Belgium; c Departement of oncology, KULeuven, Leuven, Belgium; d Department of Medical Oncology, GZA Hospitals Sint-Augustinus, Antwerp, Belgium

**Keywords:** biologicals, breast cancer, colorectal cancer, IL17, IL-23, medical dermatology, oncology, psoriasis

## Abstract

**Background:**

Biologicals targeting interleukin (IL)-17 and IL-23 improve quality of life in psoriasis and other chronic autoimmune disorders with a favorable safety profile. However, current guidelines do not recommend their use in patients with recent oncologic history due to limited evidence.

**Objective:**

To understand the impact of IL-17 and IL-23 inhibitors on cancer development, progression, and recurrence by systematically reviewing available literature.

**Methods:**

We conducted a systematic review following the Preferred Reporting Items for Systematic Reviews and Meta-Analyses guidelines.

**Results:**

Most studies investigating the use of IL-23 and IL-17 blockers did not find a higher incidence of cancer compared to the general population. One study observed no relapse in patients with a history of cancer.

**Limitations:**

The systematic review is limited due to variations in study designs and outcomes, making it difficult to achieve a comprehensive synthesis and comparison between studies. Furthermore, small sample sizes were notable.

**Conclusion:**

Preclinical studies suggest that treating psoriasis with IL-17 or IL-23 blockers is safe, also in patients witch active cancer or a history of it. Pharmacovigilance data show no increased malignancy rate in patients treated with these treatment modalities. However, data on relapse in patients with a history or active malignancy are limited.

**Capsule Summary**

• Interleukin-17 and interleukin-23 blockers improve quality of life in psoriasis with a favorable safety profile. However, guidelines do not recommend their use in patients with oncologic history.
• Most studies found no increased malignancy rates associated with biological treatments and no cancer recurrence. Therefore, these biologicals may be considered on a case-by-case basis.

## Introduction

**Fig 1:**
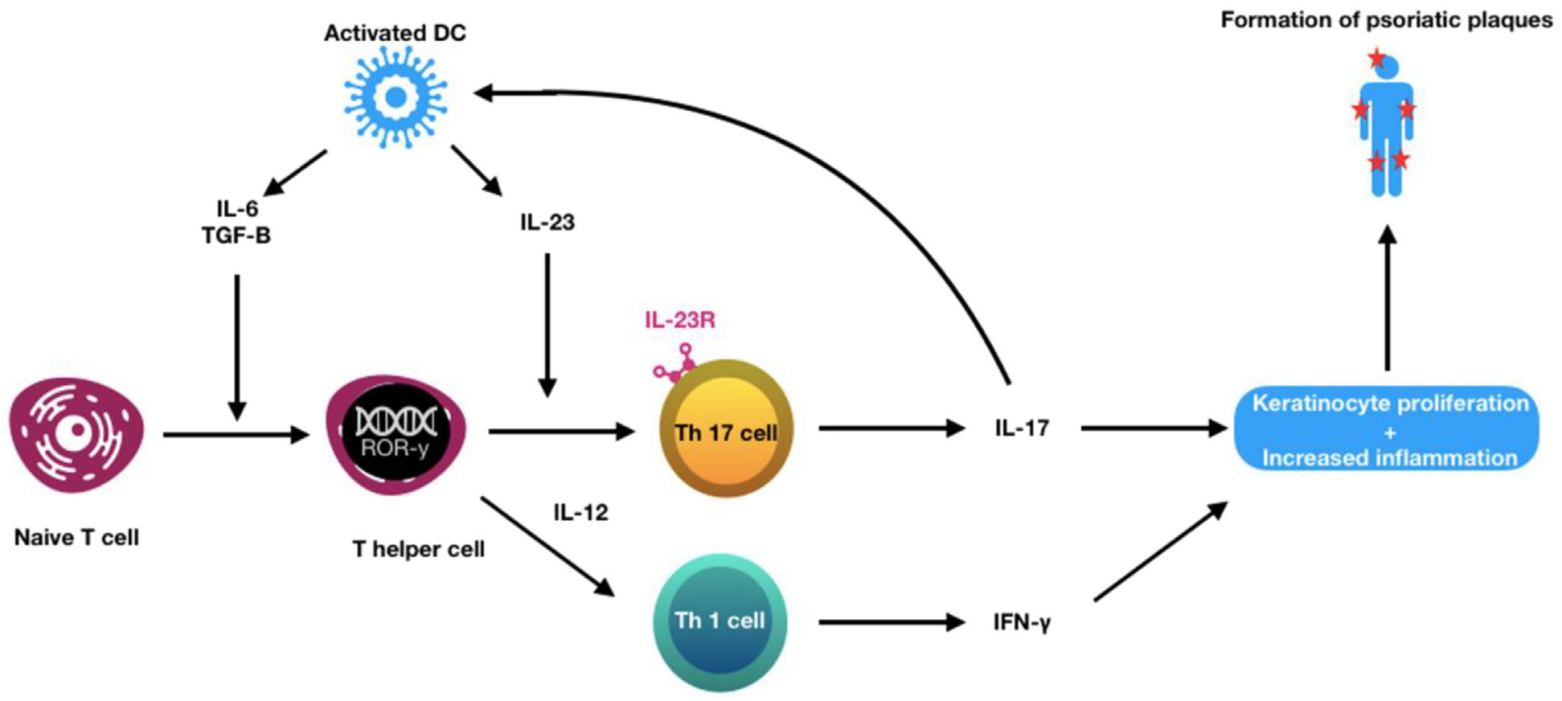
Pathogenesis of IL-23 and IL-12 in psoriasis and the cascade of cytokines involved in the differentiation of Th1 and Th17 cells. *DC*, Dendritic cell; *IFN*, interferon; *IL*, interleukin; *R*, receptor; *TGF-B*, transforming growth factor B; *Th*, T helper cell.

Psoriasis presents as a chronic autoimmune disorder, characterized primarily by erythematous plaques on the skin covered by silvery scales.^1^ It impacts 2% to 3% of the worldwide population, affecting both women and men equally. Psoriasis can manifest at any age but tends to peak between the ages of 20 and 40 years, and again between the ages of 55 and 70 years.^2^, ^3^, ^4^ It has been established that both interleukin (IL)-23 and IL-17 are crucial for sustaining psoriatic lesions.^5^ Binding of IL-23 to its receptor on memory T-helper 17 cells activates Janus kinase 2 and tyrosine kinase 2 signaling pathways, which phosphorylate and activate downstream signal transducer and activator of transcription 3 and 4 (STAT3 and STAT 4).^6^^,^^7^ This enhances the production of cytokines of the T-helper 17 family like IL-17A. Whitin keratinocytes, IL-17A interaction with IL-17 receptors triggers keratinocyte proliferation and increases the inflammatory reaction (Fig 1).^6^, ^7^, ^8^

Biologicals targeting the IL-17 family (eg, ixekizumab, secukinumab, bimekizumab, and brodalumab) and IL-23 (eg, ustekinumab, guselkumab, tildrakizumab, and risankizumab) significantly improve patients' quality of life and have a favorable safety profile.^9^, ^10^, ^11^, ^12^ Noteworthy, ongoing debate persists regarding their safety in oncology settings.

The role of IL-17 and IL-23 in the tumor microenvironment is complex. Both protumoral and antitumoral effects have been described.^13^, ^14^, ^15^, ^16^ According to Qian et al IL-17A can promote tumor progression in 4 different ways.^13^ Firstly, it could inhibit tumor apoptosis and promote tumor proliferation, by activation of downstream pathways. Secondly, IL-17A inhibits CD4+ and CD8+ T cell infiltration in some tumor types and promotes infiltration of immune cells that exhibit immunosuppressive functions, like regulatory T cells and myeloid-derived suppressor cells. By stimulating vascular endothelial growth factor and other proangiogenic factors, it promotes tumor angiogenesis. At last, IL-17A might stimulate tumor invasion and metastasis through different mechanisms like stimulating lymphangiogenesis and expression of matrix metalloproteinase 2 and 9.^13^ Donggou et al found that pre-existing skin inflammation increased tumor growth susceptibility by boosting tumor-specific IL-17 producing T cells. This susceptibility was absent in IL-17R−/− mice. Neutralizing IL-17 in mice with chemically induced skin tumors halted late-stage inflammation-driven tumor progression.^17^ Furthermore it is noteworthy that IL-23 expression, not IL-12, is elevated in human tumors, influencing local inflammation and lymphocyte infiltration in the tumor microenvironment. IL-12 fosters cytotoxic T cell infiltration, while IL-23 stimulates inflammatory responses like MMP9 upregulation and angiogenesis, but diminishes CD8 T-cell infiltration. Thus IL-23 promotes carcinogenesis not only by facilitating IL-17 production but also by inducing the expression of additional cytokines.^14^^,^^18^

In contrast to previous beliefs, an anticarcinogenic role for IL-23 and IL-17 has been described. IL-23 stimulates interferon- γ (IFN-γ) production in memory T cells, crucial for CD8+ cytotoxic T lymphocytes and antitumor responses.^19^, ^20^, ^21^ In vitro studies have demonstrated that IFN-γ exerts proapoptotic, antiangiogenic, and antiproliferative effects on tumor cells and reduced IFN-γ production is seen in cancers like breast and colorectal carcinoma.^22^ While IL-23 overexpression doesn't always induce IFN-γ, it still exhibits antitumor effects in mice lacking IFN-γ. IL-17A indirectly attracts CD4+ and CD8+ T cells to tumor sites and enhances natural killer cell activity. It also activates cytotoxic T lymphocytes and ultimately promotes neutrophil infiltration in tumor microenvironments, with neutrophils showing dual protumor and antitumor effects depending on the specific tumor environment.^13^

Data regarding the role of IL17 or IL23 in tumor processes derived from mouse models are overall reassuring: Delgado-Ramires et al found that STAT1−/− mice injected with anti-IL-17A developed significantly less tumors compared to STAT1−/− mice injected with isotype antibodies (*P* < 0.05). However, there was no difference in survival. This study suggests that suppressing IL-17A could potentially decrease tumor progression associated with STAT1 deficiency.^23^ Another study investigated breast cancer and metastasis in STAT1−/− mice, reaching similar conclusions.^23^^,^^24^ Qi et al demonstrated a significantly lower mean number of tumors in a colitis-associated cancer mouse model in the group receiving antimouse IL-17A antibody.^25^ Teng et al's study revealed that neutralizing IL-23 in mice suppresses experimental lung metastases through antitumor effects mediated by natural killer cells or CD8+ T cells.^26^ Interestingly, this contradicts previous findings suggesting that IL-23 attracts CD8+ T cells. Wight et al showed that IL-23R-deficient T regulatory cells exhibit higher sensitivity to IL-12, leading to increased IFN-γ expression and enhanced CD8+ cell infiltration, improving the antitumor response in mice.^24^ IL-23's role as an immune activator suggests an antitumor response, but its potential role in malignant cell progression creates a contradiction. The contradictory effects could be context-dependent, influenced by tissue type, cancer stage, and the host's genetic background.^20^ The predominant focus in mouse studies is on exploring anti-IL-17A therapies, although certain IL-17 inhibitors also affect additional members within the IL-17 family. There is limited literature addressing the involvement of these other family members in tumorigenesis. A recent systematic review indicates a potential protumorigenic effect of IL-17F, especially in colorectal cancer, although evidence remains conflicting. In breast cancer, findings are inconsistent.^27^

While promising outcomes are observed in mouse models, they provided controlled environments for tumor induction. Human malignancies are influenced by complex genetic and environmental factors, making direct conclusions from these models challenging.^28^^,^^29^

Breast cancer (13.3% of all cancer diagnoses) and colorectal cancer (12.7% of all cancer diagnoses) stand as the predominant malignancies in Europe. Approximately 1 in 12 women will receive a breast cancer diagnosis during their lifetime, while the cumulative risk of colorectal cancer is 1 in 22 for men and 1 in 35 for women.^30^ These statistics do not include the most prevalent human malignancies, specifically non-melanoma skin cancer (NMSC).^31^ Despite concerns surrounding the use of IL-17 and IL-23 blockers in various tumors, there exists ample confidence in their application in patients with NMSC.^32^ Hence, we found it worthwhile to examine the impact of IL-17 and IL-23 inhibitors in clinical studies pertaining to breast and colon cancer.

## Methods

This systematic review was written in accordance with the Preferred Reporting Items for Systematic Reviews and Meta-Analyses guidelines. The study protocol was registered on PROSPERO (ID: CRD42022331553).

### Review question

The primary objective of this systematic review is to comprehend the role of IL-17 and IL23 (and the impact of inhibition) on the development, progression, and recurrence of most prevalent tumors: breast and colon cancer.

### Search strategy

**Fig 2:**
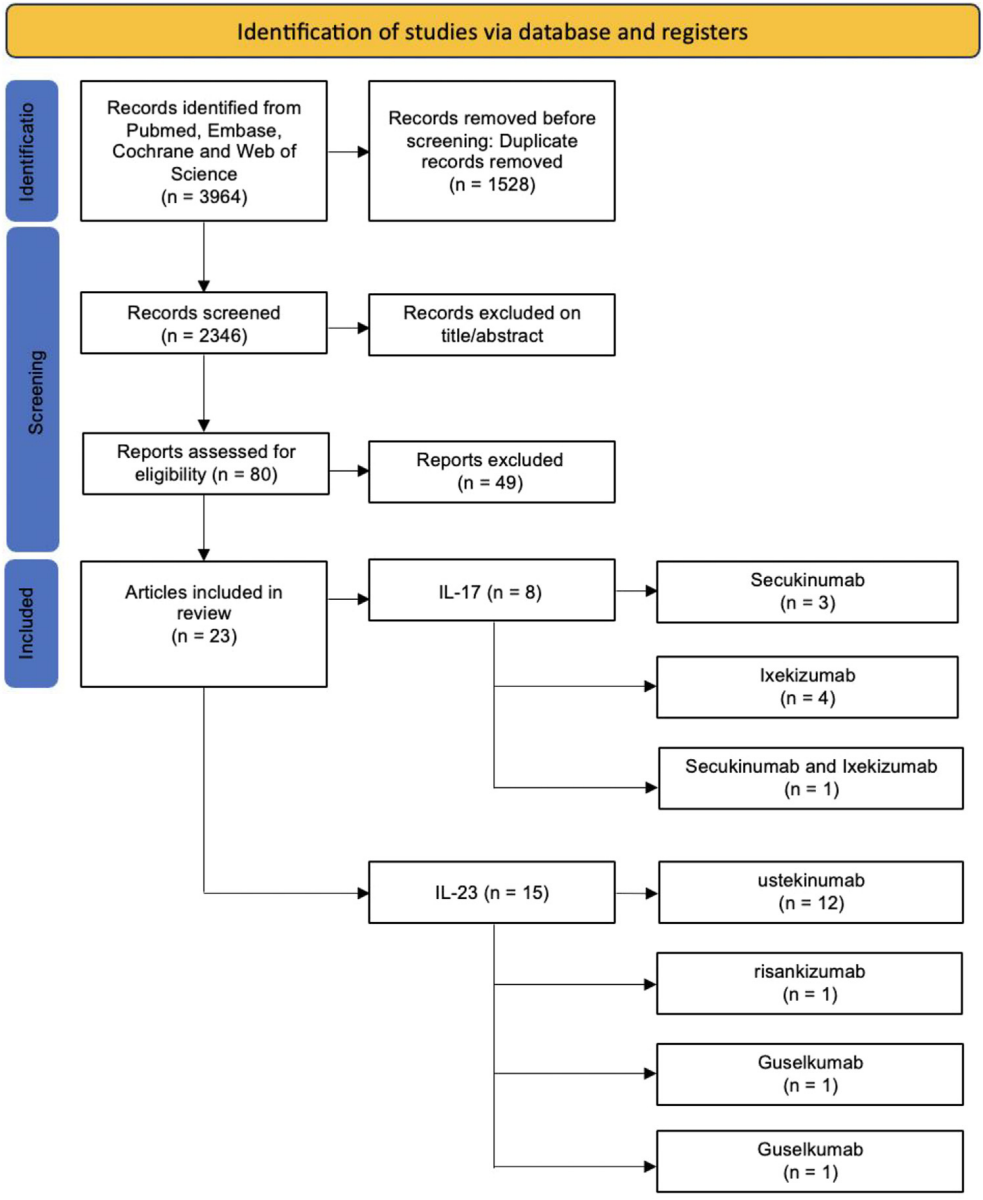
Preferred Reporting Items for Systematic Reviews and Meta-Analyses (PRISMA) literature screening flow diagram.

Our search of the relevant English literature was completed on the 14th of May 2022, using 4 different databases: PubMed, Embase, Web of Science, and Cochrane Library. For our search terms we used medical subject headings and synonyms with various spelling. Our complete search strategy is available in the Supplementary Appendix, available via Mendeley at https://data.mendeley.com/datasets/xtvmccn5yj/1 (Supplemental Appendix I. Search strategy). The selection process is illustrated in Fig 2. All the studies were released within the past decade; none of them predates 2010.

### Selection criteria

Inclusion of papers was performed by 2 independent reviewers (A.V. and A.S.) based on title and abstract using the following inclusion criteria: (1) clinical studies pertaining to the use of IL-17 or IL-23 blockers; (2) oncological setting, specifically breast and colon cancer; and (3) long-term data of more than 3 years. In case of doubt, a third reviewer (T.H.) was consulted. We excluded irrelevant articles and those with short follow-up.

### Quality assessment

The methodological quality assessment was performed by 2 independent authors for each included article according to Preferred Reporting Items for Systematic Reviews and Meta-Analyses guidelines using the National Heart, Lung and Blood Institute Study Quality Assessment tools. We applied the quality assessment tool for observational cohort and cross-sectional studies on each study (Supplementary Appendix 2, available via Mendeley at https://data.mendeley.com/datasets/xtvmccn5yj/1). In case of any disagreements, a third reviewer will decide.

## Results

### Studies on IL-23

Most studies investigating ustekinumab, did not report a higher incidence of cancer compared to the general population (Table I).^33^, ^34^, ^35^^,^^37^, ^38^, ^39^, ^40^, ^41^, ^42^, ^43^ Notably, Van Lümig et al observed no relapse in patients with a history of cancer.^42^ Magnano et al reported 9 cases of cancer at the study's conclusion, but none of them had received ustekinumab treatment.^37^ In the systematic review by Peleva et al all the studies and reviews indicated that prolonged use of IL-23 inhibitors, including ustekinumab, did not show an increased risk of cancer.^10^

**Table I:** Overview of the studies on ustekinumab

Study	Length of study in years	Number of participants	Participants treated with ustekinumab	Total cancer∗	Breast cancer†	Colon cancer†
Belinchón et al^33^	8	1938	‡	24	Not specified	Not specified
Elberdin et al^34^	10	56	19	0	0	0
Esposito et al^35^	2.5§	350	40	0	0	0
Faisal et al^36^	10‖	25	2	1	0	1
Magnano et al^37^	9	356	76	0	0	0
Papp et al^38^	5	3117	3117	54	4	5
Sandborn et al^39^	5	567	567	10	1	0
Shalom et al^40^	13	907	83	0	0	0
Staumont-Sallé et al^41^	5	305	2	0	0	0
Van Lümig et al^42^	5	173	8	0	0	0

∗ Except for skin cancer.

† In participants who were treated with ustekinumab.

‡ 13% of all the cycles of medication.

§ Years on average since the length of the study varies for every participant.

‖ On average (5.5-17 years).

In Faisal et al's study, a retrospective examination of the risk of increased cancer development after long-term treatment with biologicals in 25 individuals with familial adenomatous polyps revealed a trend, albeit nonsignificant, towards increased cancer development. The cases that developed cancer had a history of cancer.^36^

Griffiths et al's study represents a phase 3 trial where psoriasis patients were subjected to a 4-year exposure of guselkumab, adalimumab, or a placebo. Across the 3 groups, adverse events reported were generally comparable, and the incidence of serious adverse events, such as severe infections or malignancies, remained low. As the study duration increased, a gradual rise in adverse events was observed, although the overall rates remained low. This is particularly promising news, as carcinogenic processes typically require an extended period to manifest, and even after 4 years, there are still low instances of cancer development associated with guselkumab.^44^

Lebwohl et al's study explores up to 5 years safety data of tildrakizumab in psoriasis patients with and without metabolic syndrome. Notably, potential risk factors like smoking were not accounted for. In this study an increase in cancer tendency in the tildrakizumab group is observed.^45^ Papp et al's phase 3 study treated psoriasis patients with risankizumab. The most frequently reported cancers in this study, namely breast and colon cancer, were consistent with those commonly observed in the general population.^46^

### Studies on IL-17

The results of the studies regarding IL-17 are presented in Table II. Armstrong et al discovered that the incidence rate of malignancies, excluding NMSC, in patients treated with ixekizumab (0.5/100 PY) was comparable to the incidence rates in patients treated with ustekinumab (0.6/100 PY), adalimumab (0.8/100 PY), and etanercept (0.55/100 PY).^48^ Regrettably, confidence intervals were not disclosed. Combe et al concluded that longer exposure did not result in higher malignancy rates.^49^ Strober et al found incidence rates of malignancies, other than NMSC (0.5/100 PY), in patients treated with ixekizumab to be consistent with rates expected in patients with psoriasis, and comparable to etanercept during the induction period.^50^ Lebwohl et al observed patient treated with secukinumab and calculated malignancy standardized incidence ratios using the general US population as a comparison. A standardized incidence ratio of 0.99 (95% CI 0.82-1.19) indicated that the observed malignancy rates (excluding NMSC) were similar to the expected malignancy rates in the general US population.^52^ Additionally, Bellinato et al reported 10 cases of psoriasis patients a history of malignancy receiving anti-IL-17 treatment. The interval between malignancy diagnosis and the start of anti-IL-17A treatment ranged from 0 to 144 months, with a median duration of 10 months. No malignancy recurrence was detected during treatment over a median follow-up of 12 months. In a retrospective observational study of 12 patients receiving anti-IL-17 treatment, 9 in clinical remission demonstrated no recurrence, while 3 with advanced disease experienced progression.^53^ In a case report, a 50-year-old man with metastatic colon cancer, mild psoriasis, and Crohn’s disease initially responded to chemotherapy and immunotherapy (pembrolizumab). However, disease progression occurred after the introduction of secukinumab 150 mg once a week, leading the authors to suggest a potential role of IL-17 in the antitumor effects of immune checkpoint inhibitors such as pembrolizumab.^54^

**Table II:** Overview of the finding on use of interleukin-17 in long term follow-up

Study	Biological	Population	Follow up duration	No. of patients	Malignancies	Incidence rates
Zachariae et al^47^	Ixekizumab	Plaque psoriasis	>4 y	120	3 total, excl. NMSC 1 BC 1 CRC	2.5/100 PY (excl. NMSC) (0.6-10.0)
Armstrong et al^48^	Ixekizumab	Plaque psoriasis	Up to 5 y	5898 3009 >3 y 17003.4 PY	131 total, incl. 54 NMSC 5 BC 4 CRC	BCC: 0.2/100PY SCC: 0.1/100 PY Other: 0.5/100 PY
Combe et al^49^	Ixekizumab	PsA	Up to 3 y	1118 40 >3 y 1822.2 PY	13 total, incl. 8 NMSC 2 BC 0 CRC	0.9/100 PY (excl. NMSC) (0.2-3.4)
Strober et al^50^	Ixekizumab	Plaque psoriasis	Up to 5 y	4209 1166 >2 y 6480 PY	28 total, incl. 4 NMSC 2 BC 2 CRC	NMSC: 0.4/100PY Other: 0.5/100PY
Bissonnette et al^51^	Secukinumab	Plaque psoriasis and PsA	Up to 5 y	168 141 >3 y 1094,1 PY	3 total, excl. NMSC 2 BC 0 CRC	Y2: 1.2/100 PY Y5: 0.7/100 PY
Lebwohl et al^45^	Secukinumab	Plaque psoriasis, PsA and AS	Up to 5 y	14.519 285.811 PY		BCC 0.24/100 PY (0.18-0.31) SCC 0.05/100 PY (0.03-0.09) BC 0.05/100 PY (0.02-0.08) Total (incl. NMSC) 0.85/100 PY (0.74-0.98)

*AS*, Ankylosing spondylitis; *BC*, breast cancer; *BCC*, basal cell carcinoma; *CRC*, colorectal cancer; *NMSC*, non-melanoma skin cancer; *PsA*, psoriatic arthritis; *PY*, patient years; *SCC*, spinocellular carcinoma.

## Discussion

To date, it is not recommended to use IL-17 of IL-23 inhibitors in patients with a recent oncologic history according to current guidelines.^55^ This is in line with the early recommendations for anti-tumor necrosis factor-alpha therapy. However, especially patients with oncologic disease find themselves often in need of potent antipsoriatic treatments in this stressful period.

Over time, comforting data have emerged, resulting in a less clear connection between solid cancer and anti-tumor necrosis factor therapy. The role of IL-23 and IL-17 in cancer is complex. It is noteworthy that articles demonstrating the antitumor effects of IL-23 and IL-17 are often older than those describing its protumor effects.^13^, ^14^, ^15^, ^16^^,^^19^, ^20^, ^21^, ^22^, ^23^, ^24^, ^25^, ^26^, ^27^, ^28^, ^29^

In this systematic review, we reviewed clinical studies regarding IL-17 and IL-23 inhibitors in the development, progression, and recurrence of cancer in clinical studies. We focus our search on breast and colorectal cancer. Overall, available data regarding ustekinumab are reassuring: all studies except one indicate no evidence of ustekinumab being linked to increased cancer risk.^33^, ^34^, ^35^^,^^37^, ^38^, ^39^, ^40^, ^41^, ^42^ The exception is the study by Faisal et al, which focused on patients with familial adenopolyposis and Crohn's disease, conditions associated with heightened colon cancer risk.^36^ In the studies regarding tildrakizumab, we discussed the study of Lebwohl et al's, which identifies an increased risk of malignancy in the group receiving tildrakizumab. Malignancy rates were similar to previous findings in psoriasis patients and numerically elevated in those with metabolic syndrome, indicating a heightened risk for various cancers linked to metabolic syndrome. So, the question arises whether the observed effects are due to the IL-23 inhibitor or are related to factors such as metabolic syndrome.^45^ The studies concerning anti-IL-17 therapy reported reassuring data.^48^, ^49^, ^50^^,^^52^, ^53^, ^54^ Two studies examined cancer recurrence in patients treated with biologicals. Bellinato et al observed no malignancy recurrence in 10 cancer patients with psoriasis receiving anti-IL-17 during a 12-month follow-up. In a retrospective study, 9 out of 12 patients in remission showed no recurrence, while 3 with advanced disease experienced progression.^53^ Van Lümig et al found no cancer relapse in cancer patients treated with biologicals.^42^

Limitations of this systematic review are limited due to variations in study designs and outcomes, making it difficult to achieve a comprehensive synthesis and comparison between studies. Additionally, it is worth noting the small sample sizes in individual studies and the absence of randomized controlled trials examining the long-term impact of IL-17 and IL-23 inhibitors on tumor evaluation, contributing to an overall lack of statistical power. Despite the limited quality of available studies, we've presented an overview of the current evidence on IL-17 and IL-23 inhibitors in oncology, revealing a discernible trend across these studies. However, we acknowledge the need for caution in drawing definitive conclusions at this stage.

## Conclusion

Preclinical data indicate the safety of IL-17 and IL-23 inhibitors in the field of oncology. Overall, pharmacovigilance data demonstrate no heightened risk of malignancy in patients without a pre-existing history of malignancies. Nevertheless, there is a scarcity of data regarding patients with a previous history of malignancy. While European guidelines recommend topical therapy, phototherapy, or acitretin for patients with recent malignancy, the use of IL-17 and IL-23 inhibitors may be considered in individuals with active disease significantly impacting their quality of life. This consideration should ideally follow consultations with an expert oncologist tailored to each patient's unique circumstances.^55^

## Conflicts of interest

None disclosed.
